# The Kinetics Studies on Hydrolysis of Hemicellulose

**DOI:** 10.3389/fchem.2021.781291

**Published:** 2021-11-18

**Authors:** Qi Yuan, Shan Liu, Ming-Guo Ma, Xing-Xiang Ji, Sun-Eun Choi, Chuanling Si

**Affiliations:** ^1^ Engineering Research Center of Forestry Biomass Materials and Bioenergy, Research Center of Biomass Clean Utilization, Beijing Key Laboratory of Lignocellulosic Chemistry, College of Materials Science and Technology, Beijing Forestry University, Beijing, China; ^2^ State Key Laboratory of Biobased Material and Green Papermaking, Qilu University of Technology (Shandong Academy of Sciences), Jinan, China; ^3^ Department of Forest Biomaterials Engineering, College of Forest and Environmental Sciences, Gangwon National University, Chuncheon, South Korea; ^4^ Tianjin Key Laboratory of Pulp and Paper, Tianjin University of Science and Technology, Tianjin, China

**Keywords:** hemicellulose, hydrolysis, kinetics, oligosaccharides, acid, thermogravimetric analyzer

## Abstract

The kinetics studies is of great importance for the understanding of the mechanism of hemicellulose pyrolysis and expanding the applications of hemicellulose. In the past years, rapid progress has been paid on the kinetics studies of hemicellulose hydrolysis. In this article, we first introduced the hydrolysis of hemicelluloses *via* various strategies such as autohydrolysis, dilute acid hydrolysis, catalytic hydrolysis, and enzymatic hydrolysis. Then, the history of kinetic models during hemicellulose hydrolysis was summarized. Special attention was paid to the oligosaccharides as intermediates or substrates, acid as catalyst, and thermogravimetric as analyzer method during the hemicellulose hydrolysis. Furthermore, the problems and suggestions of kinetic models during hemicellulose hydrolysis was provided. It expected that this article will favor the understanding of the mechanism of hemicellulose pyrolysis.

## Introduction

Chemical kinetics explore the reaction rate and reaction mechanism of chemical processes, which is often the decisive factor in the process of chemical production (Bazant, 2013; An et al., 2019; Hu et al., 2019; Li et al., 2019; Kumar et al., 2020). In comparison with chemical thermodynamics, chemical kinetics observe the chemical reaction from a dynamic point of view and studies the required time and micro processes for the transformation of the reaction system (Real et al., 2009; Xu R. et al., 2021), whose object is a non-equilibrium dynamic system. Chemical kinetics were used to explore the mechanism of chemical reaction based on the factors, such as temperature, pressure, catalyst, and solvent (Harvey, 2007; Lu et al., 2019; Ma et al., 2020; Xu R. et al., 2020). One can control the reaction conditions, improve the rate of reaction, reduce byproducts, and improve product quality through the chemical kinetics.

Classical chemical kinetics is based on the original experimental data of concentration and time, and obtains some reaction kinetic parameters including reaction rate constant, activation energy, and pre-exponential factor. The research on the kinetics was widely carried out based on elementary reaction kinetics and active intermediates (Zarea, 2002; Liu S. et al., 2021; Liu et al., 2021; Ma et al., 2021). The reaction intermediates were detected and analyzed to understand the reaction mechanism. The chemical kinetics were widely used in chemical engineering, catalysis, pharmacy, and environment (Yang and Lee, 2005; Zazo et al., 2005; Gillespie, 2007). There are many reports on the application of kinetics in biomass pyrolysis (Ranzi et al., 2008; White et al., 2011; Mishra and Mohanty, 2018). Lignocellulosic biomass is one of the most abundant resources, which is a promising source of renewable energy (Du et al., 2019; Liu et al., 2020a; Wang H. et al., 2020; Liu et al., 2021b; Liu et al., 2021c). Lignocellulosic biomass is mainly composed of cellulose, hemicelluloses, and lignin (Liu et al., 2020b; Liu et al., 2020; Liu et al., 2021a; Du et al., 2021b; Liu et al., 2021d; Wang et al., 2021). Hemicellulose is a polysaccharide composed of different types of monosaccharides such as xylose, arabinose, galactose, and so on. The content and composition of hemicellulose vary greatly with plant species, maturity, early and late wood, cell type, and morphological position (Scheller and Ulvskov, 2010). Hemicellulose pyrolysis is a fundamental thermochemical conversion process, which is a very complex reaction (Yang et al., 2007; Du et al., 2021a; Xu T. et al., 2021). Therefore, the exact mechanism for hemicellulose pyrolysis still needs to be explored. Moreover, cellulose is the major source of fermentable sugars for the production of ethanol in biomass, which is protected by a network of lignin and hemicellulose (Girio et al., 2010; Collard and Blin, 2014; Dai et al., 2019; Yang et al., 2019; Chen et al., 2020a; Xu J. et al., 2020). The hemicellulose pyrolysis removes the protecting shield, making the cellulose more susceptible to enzymatic digestion (Chen et al., 2020b; Dai et al., 2020; Huang et al., 2020; Lin et al., 2020; Zhang et al., 2021; Zheng et al., 2021). Therefore, understanding the mechanism of hemicellulose pyrolysis by chemical kinetics is of great importance for the applications of hemicellulose.

The review paper introduced the hydrolysis of hemicelluloses, reviewed the history of kinetic models during hemicellulose hydrolysis, provided the kinetic models, and comparatively evaluated kinetic parameters. The kinetic models were influenced by many parameters such as intermediates, temperature, and catalyst. The oligosaccharides as intermediates or substrates, acid as catalyst, and thermogravimetric as analyzer method were summarized during the hemicellulose hydrolysis. We expect that this review paper would put forward the developments and applications of the hemicelluloses.

## Hydrolysis of Hemicelluloses

The structure and composition of hemicellulose vary greatly with biomass species, cell type, morphological position, and pyrolysis methods. The pyrolysis, such as autohydrolysis, dilute acid hydrolysis, catalytic hydrolysis, and enzymatic hydrolysis, is an important strategy to obtain hemicellulose and its derivatives for the fabrication of materials and chemicals (Delbecq et al., 2018). Table 1 displays the comparison of four hydrolysis methods. In the previous review article (Kapu and Trajano, 2014), Kapu and Trajano summarized the hydrolysis mechanism of polysaccharide in softwoods and bamboo in depth, and overviewed the effects of all temperature, time, acid concentration, size, reactor configuration on the hydrolysis comprehensively, and presented the types, strengths, and weaknesses of kinetic models of the hemicellulose hydrolysis. In Negahdar’s work, they reported on aqueous-phase hydrolysis of cellulose and hemicelluloses over molecular acidic catalysts in detail (Negahdar et al., 2016). Three approaches, including the simplest kinetic models, oligosaccharides as intermediates, and oligosaccharides as model compounds, were discussed to understand the reaction mechanism systematically.

**TABLE 1:** The comparison of four hydrolysis methods.

Hydrolysis methods	Advantage	Disadvantage
Autohydrolysis	Low-cost simple production	Low yield
Dilute acid hydrolysis	wide source of materials low cost	High reaction temperature, more byproducts
Catalytic hydrolysis	Short reaction time; high yield	Corrosive, environmental, and handling problems
Enzymatic hydrolysis	High specificity mild reaction conditions	Long reaction time; high requirements for equipment

The autohydrolysis and dilute acid hydrolysis methods are traditional treatments to extract hemicellulose. For example, aspen wood chip was subjected to autohydrolysis with sulfuric acid to extract hemicelluloses (Al-Dajani et al., 2009). It obtained suitable conditions for the extraction of hemicellulose using H_2_SO_4_ at a liquor to wood ratio of 4:1 for 4.5 h. In 2016, authors investigated the change in proton concentration for bamboo hemicellulose between autohydrolysis and dilute acid hydrolysis (Kapu et al., 2016). It found that all the acetyl group, ash content, initial acid concentration, and temperature affected the evolution of proton concentration during hydrolysis. The dilute sulfuric acid was used to treat three sugarcane hybrids to explore the removal of hemicellulose on the enzymatic conversion efficiency of glucan (Santos et al., 2018). It obtained the enzymatic glucan conversion of 92%–100% for post-delignification of acid-pretreated samples. A solution of hemicellulosic saccharides was obtained by non-isothermal autohydrolysis from Birch (Rivas et al., 2016). The xylan derived soluble saccharides and furfural with a yield of 80.5% based on the kinetic modeling. It converted 44.8% of substrates in furfural at 170°C with 1% sulfuric acid. Recently, Xu Y. et al. investigated the behavior of *Populus tomentosa* hemicellulose and the formation of furfural in the autohydrolysis process at 160–180°C (Xu Y. et al., 2020). The hemicellulose was converted to corresponding monosaccharides at an ultra-high hydrolysis rate. At 180°C for 2 h, it achieved the hydrolysis rate of 91, 100, 95, 58, and 37%, for xylose, rhamnose, galactose, mannose, and glucose from hemicellulose, respectively.

In addition, both acid hydrolysis and enzymatic hydrolysis were successfully developed to obtain xylooligosaccharides based on the dissolving pulp hemicellulose (Wang et al., 2018; Lin et al., 2019; Gu et al., 2020; Lin et al., 2021). It obtained the highest xylooligosaccharides yield of 45.18% from 1% sulfuric acid at 120°C for 60 min. Meanwhile, it achieved the highest xylooligosaccharides yield of 42.96% for enzymatic hydrolysis. Lopes et al. also demonstrated the separation of hemicellulose-derived sugar of xylose in acidic green ionic liquid (da Costa Lopes and Łukasik, 2018). It achieved very high recovery yields of 90.8 wt% for the ionic liquid and 98.1 wt% for xylose by alumina treatment. Obviously, the acidic green ionic liquid displayed a similar mechanism of hydrolysis of hemicellulose, compared with acid hydrolysis. Relvas*,* Morais, and Bogel-Lukasik focused on hydrolysis kinetic models of hemicellulose from wheat straw using novel high-pressure CO_2_–H_2_O method (Relvas et al., 2015a). The three accurate kinetic models of xylan conversion, arabinoxylan hydrolysis, and acetyl group hydrolysis were developed to describe the effect of CO_2_ pressure and reaction time on the intermediate compounds of xylose and arabinose. CO_2_ had an effect on the hydrolysis kinetics of hemicellulose as the fastest step of polysaccharide hydrolysis in sugars. The initial kinetic constant of the aforementioned reaction was increased by almost 40% with CO_2_, compared with the water process. Moreover, the high-pressure CO_2_–H_2_O method was also applied to selectively hydrolyze hemicellulose of wheat straw in acetic acid with a low concentration (Li M. et al., 2020). The hemicellulose selective hydrolysis was catalyzed by both carbonic acid and acetic acid. It reached the hemicellulose removal ratio of 82.3% using high-pressure acetic acid at 180°C for 1 h.

Recently, there are various reports on the enzymatic hydrolysis of hemicellulose. Ostadjoo et al., 2019 introduced the efficient enzymatic hydrolysis of hemicellulose using xylanase from *Thermomyces lanuginosus* (Ostadjoo et al., 2019). The enzymatic process enabled hydrolysis of hemicellulose to soluble oligoxylosaccharides in >70% yields. CO_2_-assisted hydrothermal method was developed for selective degradation of wheat straw hemicellulose to enhance the enzymatic hydrolysis efficiency for glucose (Wang R. et al., 2020). It obtained the significantly high efficiency of enzymatic hydrolysis due to the loose structure after hemicellulose removal. It achieved more than 72.7% of glucose after enzymatic hydrolysis, compared with merely 30.2% of untreated sample. More recently, the rational protein engineering strategy was developed to increase the catalytic efficiency for hemicellulose hydrolysis (Jaafar et al., 2021). It observed the improved enzyme catalytic reaction for insoluble substrate and the highest hydrolysis of hemicellulose by producing up to 62%, reducing sugar using variant E449D/W453Y. Dutta and Chakraborty reported the kinetics and dynamics of two-phase enzymatic hydrolysis of hemicellulose for biofuel production (Dutta and Chakraborty, 2018). The kinetic constants (Km, Vmax, Kx) assume mass transfer disguised values at 0–200 rpm. The mixing strategy increased xylose yields by 6.3–8% and reduced sugar yields by 13–20%.

In essence, acid acted as a catalyst during the acid hydrolysis of hemicellulose. In 2012, Ormsby et al. used solid acid catalysts to selective hemicellulose hydrolysis from both biochar and activated carbon (Ormsby et al., 2012). It observed an 85% conversion of xylan in 2 h for biochar and 57% in 24 h for activated carbon. The temperature increased hydrolysis reaction rate and conversion. In 2015, the solid acid SO_4_^2−^/Fe_2_O_3_ catalyst was found to selectively hydrolyze hemicellulose from wheat straw (Zhong et al., 2015). The hemicellulose hydrolysis yield of 63.5% was observed with a ratio of wheat straw to catalyst (w/w) of 1.95:1 at 142°C for 4.1 h. Dutta and Chakraborty presented a coupled experimental and theoretical framework for quantifying the two-phase enzymatic hydrolysis kinetics of hemicellulose (Dutta and Chakraborty, 2015). The xylose yield increased product inhibition and decreased reducing sugar yields. Relvas et al. used high-pressure CO_2_ as catalyst for selective hydrolysis of wheat straw hemicellulose (Relvas et al., 2015b). The CO_2_ induced the *in situ* formation of carbonic acid, obtaining a high dissolution of wheat straw hemicellulose. For high pressure CO_2_, it found a decrease in oligosaccharide content, achieving a maximum monomer sugars of 5.7 gL^−1^. In 2020, the Keggin-type molybdovanadophosphate heteropolyacids were reported as acid catalysts for the soluble mono and oligosaccharides by hydrolytic conversion of hemicellulose (Shatalov, 2020). Of the total crop xylan, 98.5% was hydrolytically converted into soluble sugars. Moreover, the solid acid sulfated zirconia was used as catalyst for the synthesis of hemicellulose hydrolysate from corncob (Wan et al., 2021). It carried out the soluble sugar concentration of 30.12 g L^−1^ with a yield of 0.33 g g^−1^ corncob and the maximum xylitol yield of 0.76 g g^−1^ from the hemicellulose hydrolysate fermented by C. Tropicalis.

## Development of Conventional Kinetic Models During Hemicellulose Hydrolysis

As early as 1945, Saeman did pioneering work in developing the kinetics of hydrolysis of wood chips in dilute acid at 170–190°C (Saeman, 1945). The author found that the activation energy was independent of the acid concentration, averaged 42,900 calories. In addition, both the increase in acid concentration and temperature resulted in an increase reaction rate. The improved Saeman model was also applied to study the kinetics of hemicellulosic hydrolysis. From then on, there are many reports on the kinetic research of hemicellulosic hydrolysis using well-known Saeman model. Moreover, more and more scholars paid attention to the kinetic models of hemicellulosic hydrolysis. In 1956, Kobayashi and Sakai explored the hydrolysis rates of pentosan of Buna (*Fagus crenata* Blume) in sulfuric acid at 74, 100, 115, 130, and 147°C (Kobayashi and Sakai, 1956). It found two stages for the hydrolysis of Buna wood pentosan with the same magnitude values for activation energy. Fagan et al. reported the acid hydrolysis kinetics of cellulose in paper refuse in 1971 (Fagan et al., 1971). In general, the hemicelluloses are more hydrolyzed than cellulose. Therefore, it is necessary for utilizing wood as chemicals and fuels to remove the hemicellulose by the pre-hydrolysis step. González et al. applied a kinetic model about the hydrolysis of hemicellulose from wheat straw in sulfuric acid at 34 and 90°C in 1986 (González et al., 1986). It yielded complete solubilization of hemicellulose to xylose and arabinose at 90°C. A two-consecutive reaction mechanism was developed about the kinetic model of acid-catalyzed hydrolysis. Conner and Lorenz used two pre-hydrolysis methods including the Iotech steam explosion process and the Stake process to explore the kinetic modeling of water and dilute acetic acid (5%) pre-hydrolysis of southern red oak wood (Conner and Lorenz, 1986). Kinetic parameters modeled the xylan removal and the occurrence of xylan oligosaccharides, free xylose, furfural, and degradation products. Thereafter, the famous biphasic model was established including “fast-reacting xylan” and “slow-reacting xylan.” The biphasic model fitted well the experimental data, compared with the Saeman model. In 1997, three different feedstocks of corn stover, poplar, and switchgrass were treated using dilute sulfuric acid (Esteghlalian et al., 1997). The authors applied first-order reactions to model monomeric constituents and degradation of the monomers by hydrolysis of hemicellulose. They used the actual acid concentration to determine the kinetic parameters of the biphasic model, predicting the percentage of as-remaining xylan and xylose. Moreover, Lu and Mosier applied the kinetic model to analyze the maleic acid-catalyzed hemicellulose hydrolysis in corn stover based on the Saeman model and biphasic model (Lu and Mosier, 2008b). It achieved the activation energy of 83.3 ± 10.3 kJ mol^−1^ for hemicellulose hydrolysis by maleic acid. It suggested low-temperature reaction conditions for monomeric xylose yield in the maleic acid-catalyzed reaction based on the Saeman model. It achieved around 80%–90% xylose yields at 100–150°C with 0.2 M maleic acid. Furthermore, Sun et al. chose five inorganic salts, such as ZnCl_2_, FeSO_4_, Fe_2_(SO_4_)_3_, FeCl_3_, and Fe(NO_3_)_3_ as catalysts on hemicellulose hydrolysis in control silage (Sun L. et al., 2011). The Saeman model fitted Fe(NO_3_)_3_ catalyzed hydrolysis for corn stover silage. It obtained the maximum yields of 81.66% for xylose and 93.36% for initial xylan. It carried out the activation energies from 44.35 to 86.14 kJ mol^−1^ for hemicellulose hydrolysis in control and from 3.11 to 34.11 kJ moL^−1^ in acid silage. Liu *et al.* reported kinetic model of dilute sulfuric acid-catalyzed hemicellulose hydrolysis in sweet sorghum bagasse for xylose production (Liu et al., 2012). It achieved the pre-exponential factors for the “easy-to-hydrolyze” fraction, the “hard-to-hydrolyze” fraction of hemicellulose, and xylose degradation of 3.53 × 10^6^, 1.80 × 10^5^, and 0.62 min^−1^, respectively, and the activation energies of 60.7, 58.1, and 14.5 kJ moL^−1^, respectively. It yielded the xylose for 60% of hemicellulose weight under 140°C for 50 min. Shi *et al.* developed the kinetics by dilute-acid pretreatment from corn Stover (Shi et al., 2017). The first-order biphasic model assumed that xylan was composed of two different fragments of fast and slow reacting fractions. The oligomers were used as intermediates in the kinetic model. It observed the low activation energies of xylan hydrolysis. Soleimani et al. explored the kinetics of hemicellulose depolymerization and decomposition in oat hull (Soleimani et al., 2018). The generation of xylose was explained by a single-phase kinetic mechanism with product decomposition (two-step sequential reaction). The single-phase mechanism was used to explain the generation of arabinose, furfural, and acetic acid. A biphasic model was applied to explain the generation of glucose in the hydrolysate due to the fast- and slow-releasing fractions into the liquid phase.

In 2003, Belkacemi and Hamoudi investigated the reaction kinetics and model of enzymatic hydrolysis of hemicellulose from corn stalk (Belkacemi and Hamoudi, 2003). A lumped model based on the Michaelis–Menten approach was used to explain the hydrolysis kinetics of two kinds of corn stalk hemicelluloses, such as xylan and heteropolymers, and three lumped species of polymeric, oligomers, and monomers. Dussan et al. also described a lumped kinetic model to simulate the pyrolysis of hemicellulose (Dussan et al., 2017). Authors proposed five new model compounds of acetylated glucuronoxylan, arabinoxylan, (galacto) glucomannan, xyloglucans, and beta-glucan toward replicating the pyrolytic reactivity of hemicellulose. Lloyd and Wyman used the depolymerization model to predict thermochemical hydrolysis of hemicellulose by dilute acid and water-only hemicellulose hydrolysis (Lloyd and Wyman, 2003). A kinetic model integrated the polymeric nature of hemicellulose to explain the polymer decomposition. In 2004, Nabarlatz, Farriol, and Montane developed a kinetic model for the autohydrolysis of xylan, which described the yields of the different reaction products and explained the chemical composition changes of the xylo-oligomers due to reaction temperature and time (Nabarlatz et al., 2004). There were two xylan fractions of three monomers (xylose, arabinose, and acetic acid) with different compositions and reactivity toward hydrolysis.

Rissanen et al. reported the extraction of hemicelluloses from spruce at 90 and 110°C (Rissanen et al., 2016). The low temperature dissolution was used to explore the early stage of extraction as the kinetic. Li et al. investigated the kinetics of hemicelluloses removal from the cold caustic extraction (Li et al., 2017). The authors indicated the hemicelluloses removal process with pseudo zero order kinetics including the bulk phase, transition phase, and residual phase. The fundamentals of hemicelluloses removal were explored by the enzymatic peeling method. Yedro et al. explored the extraction kinetics of hemicelluloses from Holm oak in subcritical water (Yedro et al., 2017; Li X. et al., 2020). It achieved the maximum yield (approximately 60%) at 170°C for 20 min. Temperature influenced significantly the hydrolysis rate of the macromolecules. Fernández et al. also applied subcritical water to extract hemicelluloses from stone pine, holm oak, and Norway spruce (Fernández et al., 2018). It observed the high activation energy of 88 kJ mol^−1^ for stone pine, 129 kJ moL^−1^ for Norway spruce, and 153 kJ moL^−1^ for holm oak. Santos-Rocha et al. presented the semi-mechanistic kinetic models of cellulose and hemicellulose for sugarcane straw (Santos-Rocha et al., 2017). The kinetic parameters were explored based on cellobiose, glucose, formic acid, and hydroxymethylfur fural (from cellulosic fraction), and xylose, arabinose, acetic acid, glucuronic acid, and furfural (from hemicellulosic fraction). Kinetic models for both cellulosic and hemicellulosic fractions degradation fitted the experimental data. Köchermann et al. explored kinetics of an aqueous organosolv hemicellulose and D-xylose conversion into furfural between 160 and 200°C using three reaction models (Köchermann et al., 2018). Kinetic models showed slight differences for D-xylose conversion and stronger deviations for furfural formation. The formation of a xylose intermediate showed the best performance. Liu F. et al. carried out the inhibitory effects of acetosyringone on xylanase activity by kinetic experiments (Liu et al., 2020). The acetosyringone obviously inhibited the activity of xylanase in a reversible and noncompetitive binding manner. He et al. developed the dissolution kinetics about the atmospheric sodium hydroxide–hydrogen peroxide extraction process of hemicellulose in bagasse pith (He et al., 2020). The activation energy of 22.19 kJ mol^−1^ indicated the time-dependent dissolution process of hemicellulose, attributing to a diffusion-controlled process. Chen et al. adopted a two-step model to predict the isothermal torrefaction kinetics of cellulose, hemicelluloses, and lignin at 200, 250, and 300°C (Chen et al., 2021). It found hemicelluloses with severe weight loss at 250°C due to the relatively weak structure. It achieved the activation energies of cellulose, hemicelluloses, and lignin in the range of 166–260, 48–55, and 59–70 kJ mol^−1^, respectively. Table 2 demonstrates the development summary of kinetic models during hemicellulose hydrolysis.

**TABLE 2:** The development summary of kinetic models during hemicellulose hydrolysis.

Kinetic models	Proposer	Reference
Saeman model	Saeman	Saeman,1945
Bidirectional model	Conner	Conner and Lorenz, (1986)
Garrote model	Garrote	Garrote et al. (1999)
Improved bidirectional model	Borrega	Borrega et al. (2011)
Bidirectional dynamic model	Tizazu	Tizazu and Moholkar, (2018)

### Effects of Modeling Hemicellulosic Hydrolysis Kinetics

During the kinetic models for hemicellulose hydrolysis, oligosaccharides are important as intermediates or substrates. In 2005, Carvalheiro et al. reported hemicellulose solubilization and xylo-oligosaccharide production by isothermal autohydrolysis treatments of brewery’s spent grain (Carvalheiro et al., 2005). Xylan and arabinan yielded oligosaccharides, monosaccharides (xylose or arabinose), furfural, and other decomposition products in consecutive reaction steps. It developed an arabinoxylan model merging the two proposed models for xylan and arabinan degradation, and including furfural formation from both pentoses. The as-proposed models provided an interpretation of the hydrolytic conversion of xylan and arabinan. In 2006, autohydrolysis of *Arundo donax* L. was reported at 150–195°C for hydrolyzing hemicelluloses to xylo-oligosaccharides with high yields of oligomers and monomers (Caparros et al., 2006). It developed a conventional kinetic model, explaining the evolution over time of the hemicelluloses and hemicellulose degradation products. Yáñez et al. assessed the suitability of autohydrolysis as a first biorefinery stage using processing of *Acacia dealbata* in aqueous media at 170–240°C (Yáñez et al., 2009). Xylan (70%) was converted into xylo-oligosaccharide at 215°C. The authors developed first-order pseudohomogeneous kinetics model, describing the *Acacia dealbata* wood solubilization as well as the autohydrolysis of the polysaccharide fractions such as glucan, xylan, arabinosyl, and acetyl substituents of hemicelluloses. In the work of Gullón, they obtained substituted xylo-oligosaccharides and solids in cellulose by the non-isothermal autohydrolysis of rye straw (Gullón et al., 2010). It found 69.2% of the initial xylan into xylo-oligosaccharide at 208°C, containing up to 22.4 g of oligosaccharide/L. It saccharified 70.6% of cellulose and 63.8% of xylan after 48 h during enzymatic hydrolysis. Kim, Kreke, and Ladisch reported reaction kinetics of xylo-oligosaccharide hydrolysis by dicarboxylic acids (Kim et al., 2013). The hydrolysis of soluble sugar oligomers followed first-order hydrolysis kinetics. The hydrolysis of xylo-oligosaccharide by dicarboxylic acids was modeled using a monophasic model based on Saeman’s pseudohomogeneous irreversible first-order reaction kinetics. Branco et al. maximized the yield of oligosaccharides by the selective hemicelluloses removal in autohydrolysis (Branco et al., 2015). It obtained a maximum of 10.4 g L^−1^ of oligosaccharides for a severity factor of 3.6. Gullón et al. reported hemicellulose solubilization and xylo-oligosaccharides production by non-isothermal autohydrolysis treatments of vine shoots (Gullón et al., 2017). The as-proposed kinetic model maximized the oligosaccharide content and minimized the generation of monosaccharides and sugar decomposition products.

In 2008, Lu et al. studied the xylose by the hydrolysis of corn stover in diluted sulfuric acid at 100°C (Lu et al., 2008a). It found the kinetic parameters of mathematical models, predicting the concentrations of xylose, glucose, and furfural in the hydrolysates. Then Jensen et al. compared the kinetic of dilute sulfuric acid hydrolysis of mixtures of hardwoods, softwood, and switchgrass (Jensen et al., 2008). It obtained an activation energy of 76.19 kJ mol^−1^ for balsam, 133.44 kJ moL^−1^ for red maple, 141.30 kJ mol^−1^ for switchgrass, 142.58 kJ mol^−1^ for aspen, and 171.20 kJ mol^−1^ for basswood. The acid hydrolysis data confirmed the validity of a pseudo first-order mixture model. After that, Xu et al. researched the kinetics of acid hydrolysis of water-soluble spruce *O*-acetyl galactoglucomannans (Xu et al., 2008). A first-order kinetic model during the acid hydrolysis was used to calculate the reaction rate constants at various pH values and temperatures. It found the activation energy of 150 kJ mol^−1^ for acid hydrolysis of spruce galactoglucomannans from the Arrhenius plot. In 2014, Rissanen et al. reported kinetics modeling of spruce hemicellulose for chemicals (Rissanen et al., 2014). A first-order model was used to perform the overall extraction data, which fitted the experimental data. It found the activation energy of about 120 kJ mol^−1^ for both chip sizes with different reaction rates and the pre-exponential factor.

In 2009, Morinelly et al. investigated the kinetics of dilute acid hydrolysis for aspen, balsam, and switchgrass at various temperatures, acid concentrations, and reaction times (Morinelly et al., 2009). The four-step kinetic model with first-order irreversible rate constants fitted the experimental data. The as-proposed kinetic model described the reaction profiles for xylose monomer by the reaction. Meanwhile, the kinetic model described the oligomer data at early reaction time. Pronyk and Mazza reported the kinetic model of hemicellulose hydrolysis from triticale straw (Pronyk and Mazza, 2010). It observed a dependency about the kinetic rate constants with flow rate. Jin et al. investigated the kinetic of hemicellulose hydrolysis of corn stover in dilute acid (Jin et al., 2011). A first-order reaction model fitted the kinetic data of hemicellulose hydrolysis. It observed over 90% of the xylose monomer yield and below 5.5% of furfural yield. It obtained the activation energy of 111.6 kJ mol^−1^ for xylose and 95.7 kJ mol^−1^ for xylose. Patwardhan, Brown, and Shanks explored the fast pyrolysis product distribution of hemicelluloses from switchgrass (Patwardhan et al., 2011). It achieved the primary pyrolysis products of the as-purified hemicellulose in decreasing abundance, such as CO_2_, formic acid, char, DAXP2, xylose, acetol, CO, 2-furaldehyde, and AXP. Shen and Wyman applied the kinetic model to explain the enhanced xylose yields from dilute sulfuric acid using reversible fast-reacting xylan and irreversible slow-reacting xylan (Shen and Wyman, 2011). The xylan removal data were simulated by a kinetic model for dilute acid and hydrothermal pretreatment of corn stover. The oligomeric xylose decomposition controlled hydrothermal autocatalytic reactions from xylan to furfural. However, monomeric xylose decomposition controlled dilute acid-catalytic reactions.

As mentioned above, acid was applied as a catalyst during the hemicellulose hydrolysis in acid system. Grénman et al. investigated the kinetics of aqueous extraction of hemicelluloses from spruce in an intensified reactor system (Grénman et al., 2011). A kinetic model fitted the experimental data. It obtained the activation energy of 135 kJ mol^−1^. In 2012, Rafiqul and Sakinah also studied the kinetic on acid hydrolysis of Meranti wood sawdust for xylose (Rafiqul and Sakinah, 2012). The kinetic parameters were used to predict the concentration of xylose, glucose, furfural, and acetic acid in the hemicellulosic hydrolysate. It obtained the 6% sulfuric acid and residence time of 20 min. Enslow and Bell reported the kinetics of Brønsted acid-catalyzed hydrolysis of hemicellulose in green 1-ethyl-3-methylimidazolium chloride (Enslow and Bell, 2012). The hemicellulose was hydrolyzed to xylose in 90% yield. In 2013, Rivas et al. researched the kinetics and manufacturing of levulinic acid from pine wood hemicelluloses *via* autohydrolysis in sulfuric acid (Rivas et al., 2013). They applied a model involved to interpret the concentration profiles including the major conversion steps such as oligomers into monosaccharides, hexoses into hydroxymethyl furfural, decomposition of this latter into levulinic and formic acids, dehydration of pentoses into furfural, and conversion of this latter into formic acid. It yielded 66% of the stoichiometric value in levulinic acid under the best conditions. In 2014, kinetic models were developed for the hydrolysis of O-acetyl-galactoglucomannan in homogeneous and heterogeneous catalysts (Salmi et al., 2014). It observed a regular kinetic behavior during the hydrolysis process using homogeneous catalysts (HCl, H_2_SO_4_, oxalic acid, and trifluoroacetic acid) and a prominent autocatalytic effect using heterogeneous cation-exchange catalysts (Amberlyst 15 and Smopex 101). The kinetic models for heterogeneous catalysts were based on the reactivity of the non-hydrolyzed sugar units and the rate constant. The kinetic model described the overall kinetics and product distribution in the hydrolysis of water soluble O-acetyl-galactoglucomannan by homogeneous and heterogeneous catalysts. A hydrogen ion catalytic kinetic model was used to predict the time-dependent behavior of xylo-oligomer, xylose, arabinose, and furfural in hydrolysates in hot water pre-extraction of moso bamboo (Hu et al., 2014). The hydrogen ion catalytic kinetic model introduced the time dependence of the hydrogen ion concentration. The authors calculated the activation energy of 98.2 kJ mol^−1^ for xylan. Moreover, Negahdar et al. investigated the kinetics of the catalytic conversion of cellobioseto sorbitol (Negahdar et al., 2014). In this work, two competing reaction pathways started from cellobiose. It obtained activation energy of 115 kJ mol^−1^ for the hydrolysis of cellobiose and 69 kJ mol^−1^ for subsequent hydrogenation of glucose, and 76 and 103 kJ mol^−1^ with overall high reaction rates at low temperatures for cellobitol formation followed by hydrolysis. Dussan et al. investigated the reaction kinetics of the major components catalyzed by formic acid in the hemicellulose fractions of D-xylose, L-arabinose, and D-glucose (Dussan et al., 2015). The reaction kinetics of solutions were predicted by these models. Chen and Liu used liquid hot water extraction to separate the hemicellulose fraction from dried distiller’s grain (Chen and Liu, 2015). A kinetic model was used to introduce the concentration of monomer, oligomer, and sugar units during acid hydrolysis. Santucci et al. evaluated a simplified pseudo-first-order kinetic model for autohydrolysis of hemicelluloses from sugarcane bagasse including all sugars, oligomers, and decomposition products from hemicelluloses (Santucci et al., 2015). Hemicellulose (61.7% of) was converted to oligomeric and monomeric sugars. It obtained an activation energy of 143.1 kJ mol^−1^ for oligomers, 158.9 kJ mol^−1^ for monomers, and 138.3 kJ mol^−1^ for sugar derivatives/decomposition compounds.

The thermogravimetric analyzer (TGA) is a very important method to explore the kinetics of biomass pyrolysis. TGA is a thermal analysis technique used to measure the change in sample mass with temperature through thermobalance and programmed temperature rise method. It can be used to get thermogravimetric curve and weight loss rate curve. By analyzing the curve, the reaction stage can be divided, the corresponding parameters can be calculated, and the pyrolysis law of the sample can be analyzed preliminarily. Rapid progress has been paid on the thermogravimetric kinetic study of hemicellulose hydrolysis. As early as 2011, Chen and Kuo developed the isothermal kinetics to explain the thermal decompositions of hemicellulose, cellulose, lignin, and xylan using TGA (Chen and Kuo, 2011). It achieved the reaction order of hemicellulose, cellulose, lignin, and xylan of 3, 1, 1, and 9, respectively, and the activation energies of 187.06, 124.42, 37.58, and 67.83 kJ mol^−1^, respectively. This model provided a good evaluation on the thermal degradations of the constituents, expect for cellulose at 300°C and hemicellulose at 275°C. Then Zhou et al. applied TGA and macro-TGA to research kinetics of pyrolysis of hemicellulose, cellulose, and lignin (Zhou et al., 2015). It found the different slow pyrolysis in the TGA and macro-TGA due to the heat transfer process, and the considerable differences of the pyrolysis and fast pyrolysis in macro-TGA. Wang et al. used TGA to explore kinetic of biomass pyrolysis using combined kinetics (Wang et al., 2016). One-step pyrolysis was developed with kinetic parameters of apparent activation energy 221.7 kJ mol^−1^ and pre-exponential factor 4.17E + 16 s^−1^. Combined kinetics three-parallel-reaction model fitted the pyrolysis experimental data. It obtained the activation energy order of three pseudo components: E_lignin_ (197.3 kJ mol^−1^) >E_cellulose_ (176.3 kJ mol^−1^) >E_hemicellulose_ (151.1 kJ mol^−1^). The pyrolysis of cellulose, hemicellulose, and lignin was comparatively studied using combined kinetics (Yeo et al., 2019). The activation energies for cellulose, hemicellulose, and lignin are 199.66, 95.39, and 174.40 kJ mol^−1^, respectively. Moreover, their group also applied TGA to investigate kinetics of biomass slow pyrolysis using distributed activation energy model, Fraser–Suzuki deconvolution, and iso-conversional method (Hu et al., 2015). The TGA technique was used to explore pyrolysis kinetics of pine wood, rice husk, and bamboo (*Bambusa chungii*). It obtained the activation energy distribution for pseudo components: E_0_ (lignin) > E_0_ (cellulose) > E_0_ (hemicelluloses), r (lignin) > r (hemicelluloses) > r (cellulose). It achieved the apparent activation energy of 162.84 ± 26.45 kJ mol^−1^ for pine wood, 168.63 ± 28.47 kJ mol^−1^ for rice husk, and 154.55 ± 26.49 kJ mol^−1^ for bamboo, respectively. A TGA was developed to explore the pyrolysis kinetic analysis of the three pseudocomponents of cellulose, hemicellulose, and lignin (Chen et al., 2017). The activation energies of cellulose, hemicellulose, and lignin pyrolysis of 112.6, 162.8, and 156.8 kJ were obtained using the modulated temperature method. The pyrolytic mechanism of cellulose, hemicellulose, and lignin was investigated using TGA (Yeo et al., 2019). The activation energies for cellulose, hemicellulose, and lignin are 199.66, 95.39, and 174.40 kJ mol^−1^, respectively. Lei et al. reported thermal pyrolysis kinetics of hemicellulose from Camellia Oleifera Shell by TGA (Lei et al., 2019). Thermal pyrolysis kinetics of Camellia Oleifera Shell hemicelluloses were investigated based on the Coats–Redfern, Flynn–Wall–Ozawa, and Kissinger–Akahira–Sunose models. The thermal pyrolysis mechanism of Camellia Oleifera Shell hemicelluloses was a one-dimensional diffusion reaction analyzed by the Coats–Redfern model. The activation energies of Camellia Oleifera Shell hemicelluloses ranged from 175.07 to 247.87 kJ mol^−1^ and from 174.74 to 252.50 kJ mol^−1^ calculated by Flynn–Wall–Ozawa and Kissinger–Akahira–Sunose, respectively. Recently, the TGA method was used to isolate hemicellulose, cellulose, and their mixture (Ding et al., 2020). The activation energy and pre-exponential factor influenced the pyrolysis process of mixture. Diez et al. reported the determination of hemicellulose, cellulose, and lignin content by TGA and pseudocomponent kinetic model (Díez et al., 2020). It obtained the good characteristic kinetic parameters of each fraction. Zhu and Zhong investigated interactions among biomass components on pyrolysis kinetics including pyrolysis experiments of individual components, synthetic biomass, and natural biomass on a TGA (Zhu and Zhong, 2020). It obtained the sharp pyrolysis behavior of cellulose with low pyrolysis reaction order (1.38), high activation energy (168.61 kJ mol^−1^), and high pre-exponential factor (3.50E + 12/s). The pyrolysis behavior of hemicellulose and lignin had a high pyrolysis reaction order (2.30, 1.51), low activation energy (126.31, 87.21 kJ mol^−1^), and low pre-exponential factor (9.67E + 09, 2.59E + 05/s). Table 3 presents the comparison for activation energy of hemicellulose hydrolysis.

**TABLE 3:** The activation energy (E/kJ mol^−1^) in comparison with hemicellulose hydrolysis.

E (kJ mol^−1^)	Reference
83.3 ± 10.3	Lu and Mosier, (2008b)
127	Sun et al. (2011b)
187.06	Chen and Kuo, (2011)
135	Grénman et al. (2011)
58.1	Liu et al. (2012)
179.84	Zhang et al. (2014)
151.1	Wang et al. (2016)
162.8	Chen et al. (2017)
95.39	Yeo et al. (2019)
147.2	Qi et al. (2020)
126.31	Zhu and Zhong, (2020)
22.19	He et al. (2020)
48–55	Chen et al. (2021)

Besides the kinetic models during hemicellulose hydrolysis, there are reports on the kinetics of dye and hydrogels. In Batzias’s work, they explored the simulation of batch and column kinetics of methylene blue and red basic 22 adsorption on mild acid hydrolyzed wheat straw as an adsorbent for wastewater dye removal (Batzias et al., 2009). Moreover, the pH-responsive hemicellulose hydrogels were prepared as carrier for controlled drug delivery (Sun et al., 2013). The swelling kinetics of the hydrogels followed Fickian diffusion process. Sun et al. synthesized stimuli-responsive porous hydrogels from wheat straw hemicellulose using CaCO_3_ as porogen for the removal of methylene blue (Sun et al., 2015b). The adsorption data was reported to be fitted to the pseudo-first-order, pseudo-second-order, and intra-particle diffusion kinetics models. The xylan/poly (acrylic acid) magnetic nanocomposite hydrogel was prepared from wheat straw xylan and Fe_3_O_4_ nanoparticles for methylene blue removal (Sun et al., 2015a). It achieved the adsorption isotherm of the Langmuir model and the pseudo-second-order kinetic model of the adsorption process. Recently, high-performance superabsorbent hydrogels were fabricated by using waste hemicelluloses lye (Liu et al., 2019). It achieved the adsorption kinetics and isotherms of the composites, and the synergy effect of polyvinyl alcohol and bentonite.

## Conclusion and Outlook

In summary, there is a long history about kinetics modeling of hemicellulose hydrolysis. Recently, it achieved the obvious development of kinetics modeling of hemicellulose hydrolysis. Various parameters, such as process, intermediates, temperature, catalyst, solvent, and systematic errors, received more attention for the kinetic models. In general, the hemicelluloses have different structures, contents, and compositions with plant species, maturity, early and late wood, cell type, and morphological position. Exploring the kinetics modeling is of great importance for understanding the mechanism of hemicellulose hydrolysis.

The kinetics modeling of hemicellulose hydrolysis still needs to be explored due to the complex structure and composition of hemicellulose. It is often carried out for the hemicellulose hydrolysis in a closed system. It is difficult to obtain the intrinsic parameters and real mechanism during the hemicellulose hydrolysis. It is also very difficult to study the kinetics of hemicellulose hydrolysis due to its complex reaction process and many influencing factors. The kinetic law based on elementary reaction conforms to the law of mass action. However, the course of most chemical reactions indicated that there existed several steps (elementary reactions) during hemicellulose hydrolysis. Therefore, accurate measurement is very important in kinetics modeling. It is urgent to establish a method for detecting active intermediates from hemicellulose. The kinetics modeling is usually derived from several assumptions. For example, the hydrolysis of xylanase reactions follows the first-order kinetic reaction equation and the hydrolysis of hemicellulose was not affected by other components in the cell wall. Arrhenius equation is a formula for the variation of chemical reaction rate constant with temperature. It should be noted that the activation energy *Ea*, as a constant independent of temperature, fits the experimental result in a certain temperature range. However, the activation energy is related to temperature in the wide temperature range or complex reaction, which is not applicable to some complex reactions. Moreover, the first premise of Arrhenius formula is that the reactions at different temperatures are consistent. However, the hemicellulose hydrolysis has different reactions at different temperatures. In short, the complexity of hemicellulose hydrolysis, closed system, and kinetics modeling itself cannot be overstated. Obviously, the development of chemical kinetics benefits from the development of modern detection methods, especially surface analysis and rapid tracking methods. It expected that the kinetics modeling of hemicellulose hydrolysis will favor the applications of hemicellulose in the near future.
